# Annotations

**Published:** 1902-05-24

**Authors:** 


### Annotations.

### The Queen of Holland.

It is with great satisfaction that we learn that the Queen of Holland has sufficiently recovered from her severe illness to be able to leave her bed. Typhoid fever, even when uncomplicated, is a sufficiently serious matter, but when complicated as in this instance by a premature confinement occurring during the progress of the fever, the condition is of necessity one of very great gravity. Whether in cases of this kind the death of the fœtus is due to disturbance of its nutrition consequent upon the morbid processes taking place in the maternal tissues, or to the fœtus itself being affected by the specific fever, may be a question, but there seems no doubt that the latter event does take place, the bacillus typhosus having been discovered in the fœtal blood and tissues. Whatever be the exact manner in which the accident is caused, it is certain that the fever and the premature confinement have a mutually injurious effect, each adding to the dangers of the other. Under such circumstances the Queen of Holland, her country, and the distinguished physicians by whom she has been attended are to be congratulated upon the happy issue to so dangerous an ordeal.

### Concurrent Small-pox and Vaccinia.

In the various discussions which take place from time to time in regard to small-pox and vaccination, a point frequently raised is as to the degree of protection which can be obtained by submitting to vaccination after exposure to infection. Some contend that vaccination done after exposure has no effect on the disease. On the other hand it is the opinion of many that vaccination begins to exercise its protective power at a very early period—at any rate by the time that the areola begins to be formed, or about the eighth day. If this be the case the vaccination must be held to have a very decided start of the small-pox, the incubation of which usually takes twelve days. It is not often possible to ascertain with sufficient exactness the date of exposure to infection in such a considerable series of cases as to be able to throw much light on the subject, but the figures concerning the vaccination of the visitors to the small-pox hospitals of the Metropolitan Asylums Board, which have recently been reported, go a long way to show that vaccination after infection is prophylactic in a very decided degree. The persons who were the subjects of the experiment were selected in an absolutely haphazard manner, being the friends of such of the patients as happened to be dangerously ill; the exposure was a single visit on a known date, the risk was approximately the same in all cases, the prophylaxis was also the same, namely, vaccination. The experiment and its results were as follow:—During the six months ending February 28th of this year the number of visitors admitted to the small-pox hospitals to see patients who were very ill amounted to 1,372. Of these 964 were protected by vaccination at the time of the visit and 7 of these, or 0·73 per cent., contracted small-pox; while of the remaining 408 persons who refused vaccination at the time of the visit, 35, or 8·58 per cent., contracted small-pox. Thus we see that taking people as they come, exposing them to the same risks and offering them the same protection, that is, a vaccination which ran concurrently with any infection they might have received, those who refused the offer suffered eleven times more than those who accepted.

### Over-Dosing with Strychnine.

We are glad to find that there are some signs of a reaction against the rather reckless dosing with strychnine which has of late years become almost a routine in the treatment of shock. There can be no doubt that we have in the hypodermic administration of strychnine a most powerful agent for good in the treatment of surgical shock as it presents itself fully formed in cases of severe accident, or of collapse as it gradually grows under our eyes in the course of prolonged operations. What is its exact *modus operandi* may not be quite clear, but at all events there is much good evidence to show that it does good, and we have not a word to say against its employment in moderate doses in cases where it may be required. We cannot but think, however, that of late the hypodermic injection of strychnine has sunk rather into a routine, and that the curious insusceptibility to its toxic action which has been so marked a feature where the collapse has been profound, and the patient has been more or less soaked with anæsthetic, has tempted some administrators to forget that they are dealing with a powerful drug which, except in the presence of such antagonistic conditions, may easily cause trouble afterwards. Certainly the cases which have been reported of clonic spasm and respiratory difficulties after its administration ought to inculcate caution in the use of the strychnine syringe—a means of making his patient "buck up" to which the modern house-surgeon has recourse with perhaps a little more freedom than may be quite judicious.

### Medical Etiquette.

The obligation which lies upon all medical practitioners not to divulge matters which have come to their knowledge in their professional capacity leads to many curious problems in regard to which it is easy to go astray if we may judge by the answers which every now and again appear in the columns of our contemporaries. The latest conundrum of the sort which we have come across touches on the question whether when a patient puts himself under the care of a new doctor the latter can demand from the former attendant information as to the treatment which he has pursued in the case. Surely it is obvious that he has no right to do anything of the sort. It seems, however, not to have occurred to some people that not only has Dr. B. no right to demand this information but that Dr. A. has no right to give it. It has been suggested by high authority that, although this information could not be demanded, it would be perfectly proper to ask for it, and that such information would in most instances be courteously supplied, all of which shows how easy it is to be drawn into backsliding if the temptation be put in such a form that obedience to one rule shall seem to involve disobedience to another. "Be ye courteous one to another" is not in the Bible, but it is a very good rule. Still we have no right, even on the plea of courtesy, to break the still higher obligation not to reveal matters which have come to us in our professional capacity—even to a "brother chip."

